# Ultrasound treatments improve germinability of soybean seeds: The key role of working frequency

**DOI:** 10.1016/j.ultsonch.2023.106434

**Published:** 2023-05-05

**Authors:** Jiahao Chen, Feng Shao, Chidimma Juliet Igbokwe, Yuqing Duan, Meihong Cai, Haile Ma, Haihui Zhang

**Affiliations:** a School of Food and Biological Engineering, Jiangsu University, Zhenjiang 212013, China; b Department of Food Science and Technology, University of Nigeria Nsukka, Enugu State, Nigeria; c Institute of Food Physical Processing, Jiangsu University, Zhenjiang 212013, China; d Nourse Pet Nutrition Jiangsu Research Institute, Zhenjiang 212009, China

**Keywords:** Dual-frequency ultrasound, Soybean, Sprouting, Water absorption, Nutrients, Antioxidant

## Abstract

In this paper, the effects of ultrasound with different frequency modes on the sprouting rate, sprouting vigor, metabolism-related enzyme activity and late nutrient accumulation in soybean were investigated, and the mechanism of dual-frequency ultrasound promoting bean sprout development was explored. The results showed that, compared with control, the sprouting time was shortened by 24 h after dual-frequency ultrasound treatment (20/60 kHz), and the longest shoot was 7.82 cm at 96 h. Meanwhile, ultrasonic treatment significantly enhanced the activities of protease, amylase, lipase and peroxidase (*p <* 0.05), particularly the phenylalanine ammonia-lyase increased by 20.50%, which not only accelerated the seed metabolism, but also led to the accumulation of phenolics (*p <* 0.05), as well as more potent antioxidant activity at later stages of sprouting. In addition, the seed coat exhibited remarkable cracks and holes after ultrasonication, resulting in accelerated water absorption. Moreover, the immobilized water in seeds increased significantly, which was beneficial to seed metabolism and later sprouting. These findings confirmed that dual-frequency ultrasound pretreatment has a great potential to be used for seed sprouting and promoting the accumulation of nutrients in bean sprouts by accelerating water absorption and increasing enzyme activity.

## Highlights

• Dual-frequency ultrasound (20/60 kHz) significantly promoted soybean germination.
• Germination improved soybean protein, polyphenol content and antioxidant activity.
• Cracks and holes in testa caused by ultrasound accelerated water absorption.
• Both abaxial side and micropyle of soybean were main water adsorption sites.
• Absorbed water improved seed viability by activating multi-metabolic enzymes.

## Graphical abstract

**Figure:**
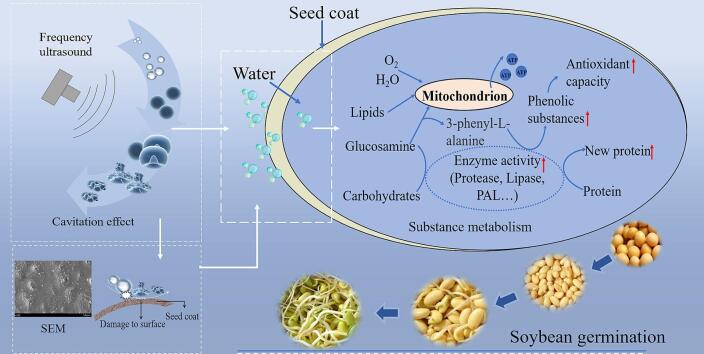

## 1 Introduction

Soybeans are crucial in the food industry due to their rich protein, oil, and bioactive chemical content [1]. In fact, soybeans and soy products are traditional foods in many nations, such as China, Japan, and Korea. Nevertheless, soy products still have some drawbacks, such as unpleasant bean flavors and antinutritional elements [1], [2]. Obviously, these unfavorable factors can significantly hinder the application in food industries. Therefore, avoiding unpleasing variables and accelerating nutrients accumulation is critical for improving the nutritional quality of soybean.

Existing data have shown that sprouted soybeans possess higher protein content and lower amounts of anti-nutritional elements (e.g., phytic acid and trypsin inhibitors) [2], [3]. Therefore, sprouting is a promising measure for improving the nutritional value and safety of soybeans. Furthermore, sprouting usually has been considered as a systematic metabolic process, including the activation of endogenous enzymes and the decomposition and/or synthesis of nutrients and bioactive compounds, such as vitamins, polyphenols, isoflavones, which are increased during sprouting [4], [5], [6]. However, rapid sprouting is difficult for dormant soybeans, which significantly hindered its industrial production [4]. In general, water absorption is the first stage in which the seed breaks dormancy and begins sprouting. Water is hydrated with stored starch and protein upon entering the seed, providing the substrate for subsequent active metabolism [7]. However, the stiff seed coat significantly hindered sprouting by preventing water from entering the seed [8], [9]. For this reason, it will become the key that how to break these physical barriers and accelerate water absorption during the early stage of soybean sprouting.

Various non-thermal processing methods, including pulsed electric fields, magnetic fields, ultraviolet light and ultrasound, have recently been used in seed-breaking dormancy and nutrient buildup [10]. Ultrasound is one of the emerging, green and sustainable non-thermal processing technologies which are widely used in food processing applications such as defoaming, cooking, drying and meat tenderization [4]. So far, the ultrasound has been applied in seed sprouting and achieved some remarkable and promising results [11]. Ampofo et al. [12] reported that the sprouting time of common beans with ultrasound treatment (40 kHz, 360 W) was reduced by 60 h while accumulating large amounts of active substances (polyphenols, anthocyanins). Similarly, Ding et al. found that the content of riboflavin and γ-aminobutyric acid in red rice increased by 60.9% and 62.5%, respectively, after ultrasound treatment (25 kHz, 16 W/L), as compared to the control group [13]. According to Chen et al. [14], the energy generated by ultrasonic vibration changed the cell wall structure, and it increased the permeability of seed cells' inner and outer membranes, which accelerated the biochemical reaction in cells and promoted seed sprouting. Chiu found that both 40 khz and 80 khz could improve seeds' hydration and sprouting rate, which might be due to the changes in seed coat structure induced by mechanical vibration caused by ultrasound [15]. In general, ultrasonic mechanism mainly included two aspects: (a) the formation of bubbles and voids induced by acoustic wave vibration and (b) the turbulence of solid induced by acoustic wave which can result in expansion and compression of solid medium (sponge effect) [16]. According to previous researches, cavitation effect can lead to the formation of microjets, contributing to improve mass transfer, and sponge effect can pump water into seed by mass transfer mentioned above [16], [17]. It has been confirmed the close relationship of ultrasonic frequency with both cavitation effect (indirect action) and sponge effect (direct action) [16]. Concretely, the intensity of sponge effect is positive linked to ultrasonic frequency, and thus an increase in the ultrasonic frequency contributed to pumping more water into seed [18]. Additionally, when the resonance frequency of bubbles is similar to ultrasonic frequency, the coupling of ultrasonic energy can reach a maximum peak, resulting in stronger cavitation effect [19]. It should be noted that extreme high of ultrasonic frequency will hinder the formation of bubbles, contributing to lower intensity of cavitation effect. Some studies have shown that single frequency ultrasound exists some disadvantages, including lesser active bubbles and uneven distribution of ultrasonic field [20], [21], [22]. Therefore, multi-mode ultrasound can form the even distribution of ultrasonic field by forming more cavitation bubbles and combined resonance effect, contributing to stronger cavitation effect and sponge effect as compared with single frequency ultrasound [19]. It has been confirmed that the acceleration of soybean sprouting may be due to the microstructural deformation of the seed coat by the cavitation effect, resulting in water entering the seed more easily [12]. Besides, sponge effect caused by ultrasound can pump more water into seed [18]. Consequently, multi-mode ultrasound may have more advantages for promoting soybean sprouting, as compared with single-frequency ultrasound. However, current research mainly focuses on the effects of ultrasonic power and single frequency on seed sprouting. The relevant information about different ultrasound frequency modes on seed sprouting is still very limited. Recently, our group has created an ultrasound device with single-frequency, dual-frequency, and triple-frequency operations, which has been used in different fields, providing the possibility for the application of multi-frequency ultrasound in the field of promoting soybean sprouting.

Therefore, the purpose of this study was to investigate the effects of different frequency patterns on the sprouting of soybean seeds, including sprouting rate, nutrients, and active substances. Furthermore, the underlying mechanism was also mainly explored from the view of water absorption. This work may provide suitable frequency ultrasound modes to accelerate seed sprouting.

## 2 Materials and methods

### 2.1 Materials

Soybean seeds were bought from Linyi (Shandong, China). The analytically pure chemical reagents, such as sodium hydroxide, sulphuric acid, hydrochloric acid, and methanol, were obtained from Sinopharm Chemical Reagent Co. Red tetrazolium (RT) and toluidine blue stain was bought from Shanghai Maclean Biochemical Technology Co.

### 2.2 Ultrasound treatment

Soybean seeds were treated with a flat-panel divergent ultrasound device developed by Jiangsu University. Soybean seeds were washed, removing bad, rotten and incomplete seeds. Selected seeds were sterilized with 75% ethanol for 5–10 min and then rinsed repeatedly with 500 mL deionized water. The seeds (1 0 0) were dispersed in an Erlenmeyer flask (500 mL) containing 200 mL of distilled water and then placed in an ultrasonic cavity (6 L), and the seeds were treated with three modes of ultrasound (60 kHz, 20/60 kHz, 20/40/60 kHz). Ultrasonic treatment was removed in the control group, and the other conditions were consistent with the experimental group. The water temperature was controlled at 25 °C ± 3 with circulating water and ice cubes. Other sonication parameters were set to power 240 W, batch ratio 5/3 (s/s), and total working time 60 min. Treated seeds were incubated at 25 °C with water sprays (10 mL) every 12 h for 96 h. After sprouting, the sprouts were collected, washed and freeze-dried, and then the dried sprout was ground and passed through an 80-mesh sieve. Finally, the sprout powder was collected for further analysis.

### 2.3 Morphological change

The following morphological alterations of soybean sprouts were observed [12], [23]:

$$\left(1\right)\;\mathrm{Sprouting percentage}\left(\%\right):\text{SP}\left(\%\right)=\frac{{\text{NS}}_{\text{t}}}{{\text{NS}}_{\text{T}}}$$

$$\left(2\right)\text{Sprouting index}:\text{SI}\left(\%\right)\text{ =}\sum {\text{N}}_{\text{Dt}}/\text{t}$$

(3) Hypocotyl (cm) and root length (cm) were measured at 96 h after sprouting with a measuring tool.

Note: *NS*_t_ is the number of sprouting seeds, *NS*_T_ is the total number of seeds, *N*_Dt_ is the number of sprouting seeds at time t, and *t* is the sprouting time.

### 2.4 Nutrients analysis

#### 2.4.1 Determination of protein, lipid, and sugar content

Protein and lipid content were determined according to the AOAC method. With minor changes, the total sugar content of bean sprouts was determined according to relevant literature [23]. The dry ground sprouts (0.5 g) were combined with 10 mL double distilled water and 1 mL HCl. The mixture was cooled and diluted with distilled water after being heated at 100 °C for 20 min, and the total sugar content was evaluated using the phenol–sulfuric acid method. The calibration standard was glucose, with a linear concentration range of 0 mg/L to 100 mg/L.

#### 2.4.2 Total phenols and total flavonoids determination

##### 2.4.2.1 Preparation of the extracts

The extracts were made using the method of Marathe et al. with minor alterations [24]. The sprout powder (1 g) was combined with 10 mL of 80% aqueous methanol solution (V/V) and stirred continuously at room temperature for 2 h (using an orbital shaker at 150 rpm). After incubation, the mixture was centrifuged for 20 min at 8000 rpm. The supernatant was filtered through filter paper, and the residue was re-extracted for 1 h in 15 mL of 80% methanol. With 80% methanol, the pooled extract was raised to 30 mL. Until analysis, all extracts are stored at −20 °C.

##### 2.4.2.2 Content determination

The total phenol and flavone content of soybean sprouts were measured using the Bueno et al. method [25].

### 2.5 Antioxidant activity assay

The DPPH (2,2-diphenyl-1-picrylhydrazyl) radical scavenging activity of bean sprouts was determined as documented by Ampofo et al. with minor modification [12]. Briefly, 100 µL of bean sprout extract was combined with 100 µL of 0.1 mM DPPH solution, vortexed for 1 min, and non-exposed to light for 30 min, and the absorbance at 517 nm was recorded. The following equation was used to calculate the results:

$$Scavenging\;activity\;\left(\%\right)\;=\left(1-\frac{A_{1}-A_{2}}{A_{0}}\right)\times \%$$

*A*_1_: sample and DPPH(ABTS), *A*_2_: sample and distilled water, *A*_0_: distilled water and DPPH.

### 2.6 Phenylalanine ammonia-lyase activity assay

The extraction of L-phenylalanine ammonia-lyase (PAL) was referred to the method of Ampofo et al. [12]. The reaction mixture (100 µL of enzyme extract, 1 mL of 0.02 M L-phenylalanine and 2.4 mL of PAL extraction buffer) was incubated for 60 min at 30 °C. Subsequently, the reaction was terminated with 0.5 mL of 10% Trichloroacetic acid (TCA). The reaction was centrifuged (10000 g, 10 min) at 4 °C and the absorbance was measured at 290 nm with UV/vis spectrophotometer (Munich, Germany). One unit was defined as 0.1 of the absorption change value at 290 nm caused by protein (1 mg/mL) per minute. Results were expressed as U/mg of protein.

### 2.7 Water absorption by seeds

#### 2.7.1 Determination of water absorption and water absorption rate of seeds

Fifty seeds were selected and placed in a conical flask containing 100 mL of water for ultrasonic treatment. The soaked beans were weighed *via* analytical balance at every ten minutes. It should be noted that water present in the surface of soybean needs to remove before weight. The following equation was used to calculate the percentage of water absorbed and their water absorption rate.

Water absorption percentage:

$${\text{W}}_{\text{a}}\left(\%\right)=\left({\text{W}}_{\text{n}}-{\text{W}}_{\text{0}}\right)/{\text{W}}_{\text{0}}$$

Water absorbing rate:

$$W_{r}\left(g/\min\right)=\left(W_{n}-W_{n-1}\right)/t$$

*W*_0_: Seed weight before ultrasound, *W*_n_: Weight of seeds at time n after soaking, *W*_n-1_: The mass of the seeds at the previous time points of a certain time points of immersion, *t*: The interval time (10 min).

#### 2.7.2 Determination of expansion percentage and expansion rate

The seeds were placed in the measuring cylinder with 20 mL of oil after sonication and the increase of oil volume to reflect the expansion of the seeds after ultrasonic treatment. The measurement was performed once every ten minutes, and the total measurement period was 60 min. The control group without ultrasound, the rest of the conditions remained the same as the ultrasound group. The following formula was used to obtain the expansion percentage and expansion rate:

Expansion percentage:

$$E_{p}\left(\%\right)=\left(V_{n}-V_{o}\right)/V_{o}$$

Expansion rate:

$$E_{r}\left(\%\right)=\left(V_{n}-V_{n-1}\right)/t$$

*V*_o_: volume of oil before immersion; *V*_n_: volume of oil at a point in time after seed immersion; *V*_n-1_: volume of oil increase at a point in time before seed immersion; *t*: interval time (10 min).

#### 2.7.3 Low-field NMR determination of seed internal moisture

An MRI analysis (NMI20-060VJS-I) was used to evaluate water distribution within the seeds. The temperature of the sample chamber was 32.00 ± 0.01 °C. The CPMG (Carr-PurcellMeiboom-Gill) pulse sequence was used to measure the samples. The CPMG sequence parameters were as follows: number of sampling points (TD):700408, number of echoes (NECH):17000, echo time (TE):0.206 ms, and sampling rate:200. All samples were measured three times.

### 2.8 Seed viability test

The method of Zhou et al. is used and significantly modified [26]. Briefly, 45 seeds were soaked in 0.5% TTC solution (100 mL). After that, selected seeds were treated with ultrasound. Finally, the number of red-stained seeds was recorded, and their images were also taken to analyze seed viability.

### 2.9 Initial site of water entry into seeds

Fifty soybean seeds were soaked in a 0.5% saturated toluidine blue solution. The solution was treated in an ultrasound unit at 25 °C. The seeds were sectioned longitudinally with a disposable slicer blade, and the entire surface was examined under the stereomicroscope. The sectioning staining was examined under a stereomicroscope to determine the initial location of water entry into the seed [27].

### 2.10 Surface structure

Soybean seeds that had been sonicated were removed and chopped into little pieces (5 cm). The seed coat was fixed in a 2.5% glutaraldehyde solution for 12 h before being rinsed three times for 15 min, each with 0.1 mol/L phosphate buffer (pH 7.4). Then, for 30 min each time, gradient dehydration was performed with 30% to 100% alcohol, and anhydrous ethanol was replaced by isoamyl acetate in volume ratios of 1:1 and 1:2. The coatings were cured, sprayed with gold, and imaged using scanning electron microscopy.

### 2.11 Statistical analysis

The data were presented as mean ± standard deviation. SPSS software was used for statistical analysis. The Tukey test with an alpha level of 0.05 was used to evaluate differences between sample means using one-way ANOVA.

## 3 Results

### 3.1 The effect of single ultrasonic frequency on the sprouting percentage of soybean seeds

**Fig. 1:**
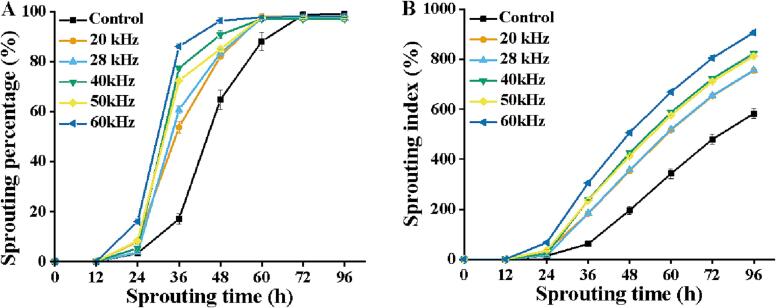
Sprouting percentage (A), Sprouting index (B) of soybean seeds under the single frequency ultrasound. Results are presented as mean ± SD of three independent experiments.

As shown in Fig. 1A, ultrasonic treatment significantly improved the sprouting percentage of soybean seeds compared with the control group during the sprouting of 24 to 60 h (*p* < 0.05). Especially, after 48 h of sprouting, treatment with 60 kHz of ultrasound showed maximum sprouting percentage, which was 13.33%, 6.24%, 15.60%, 17.47%, and 49.02% higher than that of the other ultrasonic treatments (50 kHz, 40 kHz, 28 kHz, 20 kHz) and the control treatment, respectively. Similarly, the results (Fig. 1B) revealed that seed viability was the highest at 60 kHz, which was ascribed to a greater sprouting index, as compared to the other ultrasounds and control group (*p* < 0.05) [12]. The sponge effect of different ultrasonic frequencies causes varying mass transfer effects. In general, the intensity of the sponge effect is enhanced by the increase in ultrasound frequency [16]. Compared with the other ultrasound frequencies, ultrasound of 60 kHz might promote more water absorption by soybean seeds, which enhanced the cellular respiration and increased the sprouting rates, indicating that 60 kHz is a sensitive frequency for soybean sprouting. Based on this, the ultrasonic frequency of 60 kHz was utilized as a base point for the following investigations.

### 3.2 Effects of different ultrasound modes on the sprouting and morphology of soybean

Based on the results of 3.1, the multi-frequency ultrasound equipment developed by our research group was utilized to analyze the feasibility of further improvement on the effectiveness of single-frequency ultrasound (60 kHz) by combining other ultrasound frequencies like 20 and 40 kHz.

**Fig. 2:**
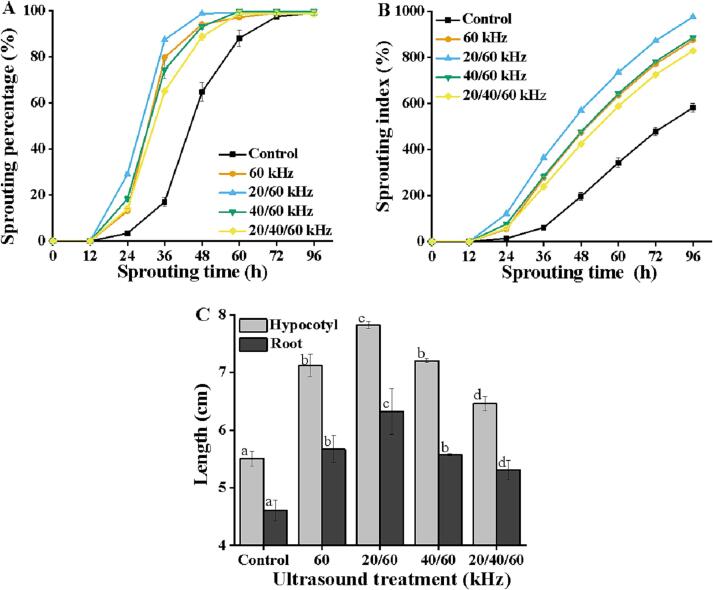
Sprouting percentage (A), Sprouting index (B), Hypocotyl and root length (C) of soybean seeds. Results are presented as mean ± SD of three independent experiments. Different characters (a–d) on the top of the column indicate significant (*p* ≤ 0.05) differences among samples treated under different ultrasound levels.

Fig. 2 showed that all ultrasonic modes could significantly accelerate the sprouting of soybean seeds above those of control. After ultrasonication, soybeans had a 48.00 ∼ 73.33 % (36 h) higher sprouting percentage and a 42.54 ∼ 67.80% (96 h) higher sprouting index compared to the control group. More precisely, dual-frequency ultrasound at 20/60 kHz resulted in the fastest sprouting. A 100% sprouting percentage was obtained at 48 h, which was 12 h earlier than single-frequency ultrasound (60 kHz). Additionally, it should be noted that dual-frequency ultrasound of 40/60 kHz did not differ much from the single-frequency ultrasound on sprouting (*p >* 0.05). Previous research confirmed the positive relationship of ultrasonic frequency with sponge effect and negative relationship with cavitation effect [16], [20]. Thereby, compared with 40 kHz, treatment with ultrasound at 20 kHz shows stronger cavitation effect, while ultrasound at 60 kHz exhibits stronger sponge effect. Dual-frequency ultrasound (20/60 kHz) combines the characteristics of two frequencies (20 kHz and 60 kHz), leading to stronger sponge effect and cavitation effect, which may be the main reason for its acceleration of soybean sprouting. Furthermore, the results in Fig. 2A and B reveal that triple-frequency ultrasound (20/40/60 kHz) only caused higher effects than the control, which might be related to the superimposition of too many frequencies damaging the seed microstructure [22].

In addition, the hypocotyl and root lengths were measured after 96 h of sprouting, shown in Fig. 2C. Similar to prior findings, dual-frequency ultrasound at 20/60 kHz provided the longest shoots (7.82 cm) and roots (6.32 cm), which were 2.32 cm and 1.72 cm longer than the control group. Previous research has found that the beneficial development of bean sprouts can be attributable to accelerated cell division and increased endogenous hormone expression after ultrasound [12]. Overall, different frequency patterns had varying effects on sprouting. Dual-frequency ultrasound of 20/60 kHz offers a demonstrable benefit in stimulating sprouting.

### 3.3 Antioxidant activity

According to the study of Ampofo et al. [12], antioxidant capacity is the ability of the compound to shield biological systems from the detrimental effects of oxidative stress. The antioxidant capacity of soybean sprouts was dramatically increased following ultrasound compared to the control group (shown in Table 1). The maximum ability to scavenge DPPH radical formation (72.60%) was observed for the dual-frequency ultrasound (20/60 kHz) treatment at 96 h of sprouting, and this was significantly higher compared to the other ultrasonic frequency (60 kHz, 40/60 kHz, 20/40/60 kHz) and control treatments for the same sprouting time by 3.74%, 9.68%, 9.82%, and 19.41%, respectively. Antioxidant chemicals (such as flavonoids and phenolic acids) quench these harmful radicals by supplying hydrogen and electron atoms [3]. Generally, phenolic compounds with high numbers of hydroxyl groups can exhibit more potent antioxidant activity [12]. As a result, dual-frequency ultrasound (20/60 kHz) may improve antioxidant activity of soybean sprouts by altering the number and structure of free phenols. Moreover, the phenylalanine ammonia-lyase (PAL) has been considered as key enzyme during the phenolic metabolism, and dual-frequency ultrasound (20/60 kHz) may increase the activity of PAL, contributing to more polyphenolic accumulation [4], [12]. Therefore, dual-frequency ultrasound (20/60 kHz) can significantly improve the antioxidant properties of soybean during sprouting. Overall, dual-frequency ultrasound of 20/60 kHz is superior to other multi-frequency ultrasounds (40/60 kHz, 20/40/60 kHz) regarding sprouting percentage and antioxidant capacity.

**Table 1:** DPPH (%) of soybean sprouts elicited with ultrasound during sprouting. Data are presented as mean ± SD of three independent experiments. Different superscript letters within rows indicate significant differences (*p* < 0.05).

Sprouting time (h)	Ultrasound treatment				
	Control	60 kHz	20/60 kHz	40/60 kHz	20/40/60 kHz
24	35.18 ± 3.75^a^	45.45 ± 0.54^b^	43.10 ± 3.34^bc^	46.48 ± 0.28^b^	41.15 ± 0.92^c^
48	36.44 ± 1.90^a^	40.15 ± 1.21^b^	42.49 ± 0.21^c^	36.91 ± 0.76^a^	38.80 ± 1.72^a^
72	54.92 ± 4.34^a^	55.19 ± 1.38^a^	68.25 ± 2.23^b^	65.10 ± 1.50^c^	56.09 ± 0.94^a^
96	60.80 ± 0.26^a^	69.98 ± 0.76^b^	72.60 ± 0.83^c^	66.19 ± 1.48^d^	66.11 ± 0.50^d^

### 3.4 Chemical composition of sprouts

To further explore the reason for the high efficiency of dual-frequency ultrasound at 20/60 kHz, a single-frequency ultrasound and a non-ultrasound group were utilized as controls in the following investigations.

#### 3.4.1 Protein, lipids and sugars

Table 2 described the variations in nutrients in soya sprouts. After 96 h of sprouting, the highest protein accumulation with dual-frequency sonication (20/60 kHz) was 1.64% and 4.54% higher than the single-frequency sonication (60 kHz) and control groups, respectively. Furthermore, as compared to the control group, ultrasound treatment dramatically lowered the levels of lipids and carbohydrates. According to Yang et al., lipid and sugar degradation were employed to generate energy for protein synthesis during sprouting [23]. Moreover, the effect of ultrasound on enzyme activity was evaluated (Supplementary material) and the results showed that ultrasonic stimulation significantly increased the activities of soybean seed protease, lipase and amylase above the control group (*p <* 0.05). Overall, the activity of protease, amylase and lipase were significantly improved after dual-frequency ultrasound of 20/60 kHz treatment, which means that faster internal metabolism. During sprouting, lipid and carbohydrates were usually used to support the synthesis of protein [4], [23]. Obviously, application of dual-frequency ultrasound (20/60 kHz) significantly accelerates above metabolism process.

**Table 2:** Changes in protein, lipid and sugar contents of soybean sprouts after ultrasound treatment. Data are presented as mean ± SD of three independent experiments. Different superscript letters within a column indicate significant differences (*p* < 0.05).

Nutrients (%)	Ultrasound Treatment (kHz)	Sprouting time (h)			
		24	48	72	96
Protein	Control	27.74 ± 0.53^a^	28.98 ± 0.15^a^	29.05 ± 0.65^a^	29.71 ± 0.36^a^
	60	28.66 ± 0.26^b^	29.44 ± 0.15^b^	29.95 ± 0.25^b^	30.56 ± 0.33^b^
	20/60	28.45 ± 0.06^b^	30.22 ± 0.47^c^	31.20 ± 0.17^c^	31.06 ± 0.19^c^
Lipid	Control	20.34 ± 0.13^a^	19.42 ± 0.20^a^	18.49 ± 0.12^a^	16.50 ± 0.46^a^
	60	20.28 ± 0.41^a^	19.00 ± 0.58^ab^	16.90 ± 0.33^b^	14.52 ± 0.24^b^
	20/60	19.74 ± 0.32^a^	18.09 ± 1.00^b^	16.60 ± 0.29^b^	14.65 ± 0.50^b^
Sugar	Control	14.80 ± 1.08^a^	13.89 ± 0.57^a^	10.37 ± 1.58^a^	7.68 ± 0.88^a^
	60	12.65 ± 0.35^b^	11.47 ± 0.92^b^	7.73 ± 0.87^b^	7.44 ± 0.76^a^
	20/60	12.09 ± 0.50^b^	9.08 ± 1.24^b^	7.28 ± 0.49^b^	5.50 ± 0.68^b^

#### 3.4.2 Total phenols, total flavonoids and phenylalanine ammonia-lyase (PAL)

The phenylalanine ammonia-lyase (PAL) is rated as the key enzyme responsible for triggering the phenylpropanoid pathway, a metabolic pathway for phenolic biosynthesis [28]. During sprouting, ultrasound treatment significantly enhanced PAL activity. After 96 h of sprouting, PAL activity under dual-frequency ultrasound was 7.03% and 20.50% higher than that in single-frequency ultrasound group and control group, respectively (Table 3). Yang et al. [23] found that ultrasonic treatment can improve the activity of PAL and TAL, attributed to considerable signaling of phenylalanine and tyrosine (PAL and TAL substrates, respectively) during sprouting.

**Table 3:** Total phenols, total flavonoids and PAL activity of soybeans after ultrasound treatment. Data are presented as mean ± SD of three independent experiments. Different superscript letters within a column indicate significant differences (*p* < 0.05).

Nutrients	Ultrasound Treatment (kHz)	Sprouting time (h)			
		24	48	72	96
Total phenols (mg/100 g)	Control	59.61 ± 1.70^a^	68.89 ± 0.73^a^	101.80 ± 2.57^a^	124.09 ± 0.46^a^
	60	63.46 ± 1.72^b^	79.48 ± 1.54^b^	102.87 ± 1.59^a^	126.86 ± 0.22^b^
	20/60	71.11 ± 3.24^c^	70.80 ± 0.70^a^	107.64 ± 3.82^b^	138.40 ± 1.54^c^
Total flavonoids (mg/100 g)	Control	60.97 ± 0.98^a^	92.46 ± 3.77^a^	101.72 ± 3.49^a^	103.80 ± 1.23a
	60	73.11 ± 0.23^b^	96.02 ± 3.58^a^	101.09 ± 3.97^a^	107.08 ± 1.19^b^
	20/60	70.90 ± 0.97^c^	105.58 ± 1.39^b^	96.91 ± 3.61^a^	110.76 ± 1.34^c^
PAL (U/mg protein)	Control	19.95 ± 0.75^a^	21.31 ± 0.66^a^	22.93 ± 0.13^a^	27.72 ± 0.51^a^
	60	20.65 ± 0.30^a^	24.36 ± 0.11^b^	29.33 ± 0.70^b^	31.15 ± 0.11^b^
	20/60	19.21 ± 0.58^a^	25.79 ± 0.60^b^	30.35 ± 0.20^b^	33.34 ± 1.04^c^

The significant increase in PAL activity triggered the accumulation of phenolics during soybean sprouting. The highest total phenol content was obtained with dual-frequency sonication after 96 h of sprouting, with a significant increase of 8.78% and 11.53%, respectively, compared to single-frequency sonication and control treatment at the same time. Similarly, after 96 h of sprouting, higher value was clearly observed in the total flavonoid content of soybean with dual-frequency ultrasonic treatment, as compared with single-frequency ultrasound and control group (*p <* 0.05). Similar results also were obtained in red bean sprouting [15]. In that study, the authors discovered a considerable rise in the flavonoid and polyphenol content of red beans following sonication. The results indicated that dual-frequency ultrasound has a considerable advantage regarding active substance accumulation, which is advantageous for producing active substance-enriched bean sprouts.

### 3.5 Effect of ultrasonic frequency on water uptake by soybean seeds

#### 3.5.1 Water absorption and volume change of seeds

**Fig. 3:**
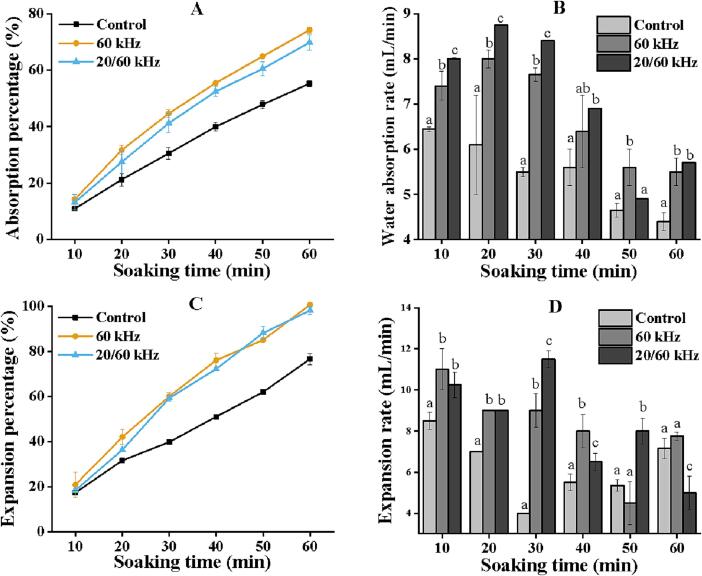
Water absorption and volume changes of soybean seeds after ultrasound treatment. Different characters (a–c) on the top of the column indicate significant (*p* ≤ 0.05) differences among samples treated under different ultrasound levels. Notes: A: Absorption percentage of water, B: Water absorption rate, C: Expansion percentage, and D: Expansion rate.

Physical dormancy is especially common in legumes [29]. The dormant seed relies on appropriate water availability for embryo development to resume [30]. When a seed grows, however, an impenetrable covering or testa forms, which blocks the intake of water required to commence embryonic development [31]. As a result, tests were carried out to investigate the influence of ultrasound on seed water absorption. Fig. 3A and C indicate that applying ultrasound to the seeds enhances the percentage and rate of water absorption. Specifically, single and dual-frequency ultrasound absorption rates were consistently higher than the control group during seed soak *(p <* 0.05). However, there was no significant difference in water absorption between dual-frequency and single-frequency ultrasound except at 60 min of soak. Some studies have reported that ultrasound causes the solid medium to contract and inflate like a sponge alternately and the “ sponge effect” is responsible for the faster mass transfer of water [16]. A similar result has been observed in studies with brown rice [32] and mung beans [9]. Fig. 3C showed that after ultrasonic treatment, the water absorption rate increased and then declined, substantially different from the control group. This suggested that ultrasound considerably influences the early stages of water absorption. The highest absorption rate was achieved at 20 min with dual-frequency ultrasound, which was 9.38% and 43.44% higher than single-frequency ultrasound and the control group. The sponge effect induced by ultrasound is frequency-dependent [18], and multi-frequency ultrasound also induces a more substantial cavitation effect and releases more energy, which can be applied to explain a higher water absorption rate after dual-frequency ultrasound treatment.

Additionally, following sonication, the percentage expansion of the seeds rose dramatically. However, no significant difference was between frequencies (Fig. 3C). The water then hydrates with the hydrophilic material in the seed, causing the seed to swell [32], [33]. Fig. 3D showed the expansion rate of soybean seeds, with the ultrasonically treated seeds showing fluctuating variations and being more significant than the control group until 40 min.

Overall, ultrasound treatment contributed to faster water absorption and seed expansion. In the early stages, the absorption rate of seeds using dual-frequency ultrasound was significantly higher than that of single-frequency ultrasound (Fig. 3B). The rapid water absorption resulted in a considerable rise in seed expansion at 30 min (Fig. 3D). Rapid water absorption with dual-frequency ultrasound can cause seed respiration to resume early, giving an advantage for faster metabolism before sprouting.

#### 3.5.2 Water distribution of seeds

**Fig. 4:**
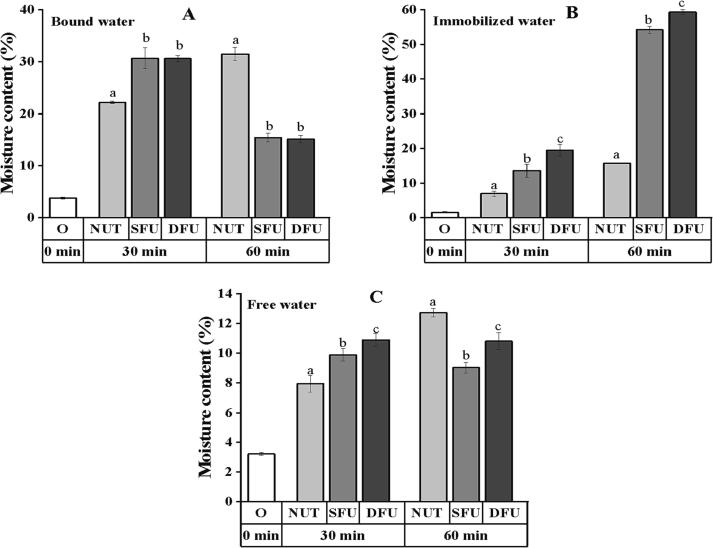
Moisture distribution of each experimental group after 30 min and 60 min of ultrasonic treatment. Different letters indicate significant differences (*p* ≤ 0.05). Notes: A: Bound water, B: Immobilized water, C: Free water.

The moisture distribution throughout the seed was monitored by low-field NMR. The bound water (Fig. 4A) of the seeds rose considerably after 30 min of sonication compared to the control(*p* < 0.05), but there was no significant difference between the two sonication groups. Meanwhile, at 30 min, the immobilized water (Fig. 4B) was 43.70% and 181.79% higher with dual-frequency ultrasound than with single-frequency ultrasound and the control group, respectively. Water is hydrated with stored starch and protein upon entering the seed, providing the substrate for active metabolism [33], [34]. The vibration induced by ultrasound (sponge effect) increased the binding degree of moisture within the seed [9], [16], resulting in a considerable increase of bound and immobilized water. Similarly, studies have demonstrated that in order to avoid osmotic damage, seeds increase the amount of immobilized water during water uptake, and the experimental results (Fig. 4B) appear to validate this phenomenon [7], [35].

In addition, the immobilized water content of ultrasonic seed increases after 60 min (Fig. 4B) while the bound water content decreases (Fig. 4A). The results demonstrate the partial conversion of bound water to immobilized water as a result of ultrasonic stimulation. However, the control group seemed to delay this change as its bound water and free water content only reached 31.44% and 12.73% at 60 min, and the ultrasound group completed it at 30 min.

### 3.6 Seed viability identification

**Fig. 5:**
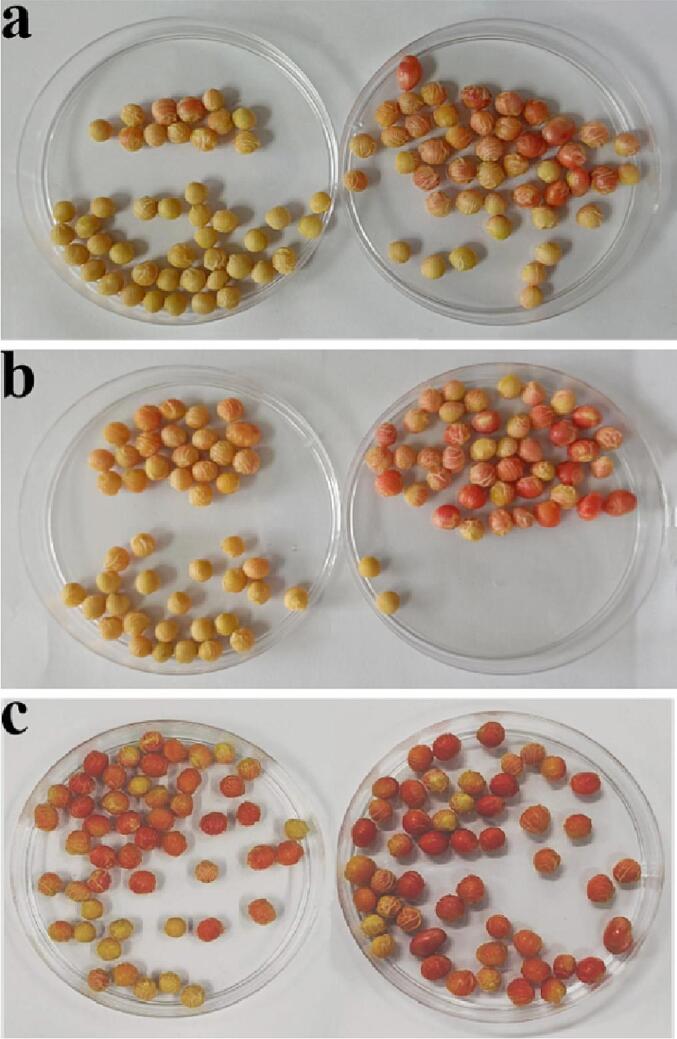
Recorded are the phenomena of staining seeds immersed in TTC. Note: a: Control group, b: single-frequency sonication, c: double-frequency sonication. Left Petri dish: seeds soaked for 30 min, right Petri dish, soaked for 60 min.

Due to the fact that 2,3,5-triphenyl tetrazolium chloride (TTC) can be reduced to red by dehydrogenase, it is usually applied to evaluate respiratory activity in cells. In general, the respiration intensity of soybean is closely related to its metabolic intensity, therefore this study used TTC to visually characterize respiration during seed sprouting, confirming the promoting role of ultrasound in soybean metabolism [36]. The significant benefits of ultrasonically treated seeds depend on the degree and color of staining (shown in Fig. 5). The number of red seeds exceeded 70% at 30 min with dual-frequency sonication, considerably above that of single-frequency sonication (46.7%) and the control group (26.7%). The results showed that seed vigor was stimulated sooner in the dual-frequency ultrasound treatment, providing a significant benefit for fast seed development.

### 3.7 Initial site of water entry into seeds

**Fig. 6:**
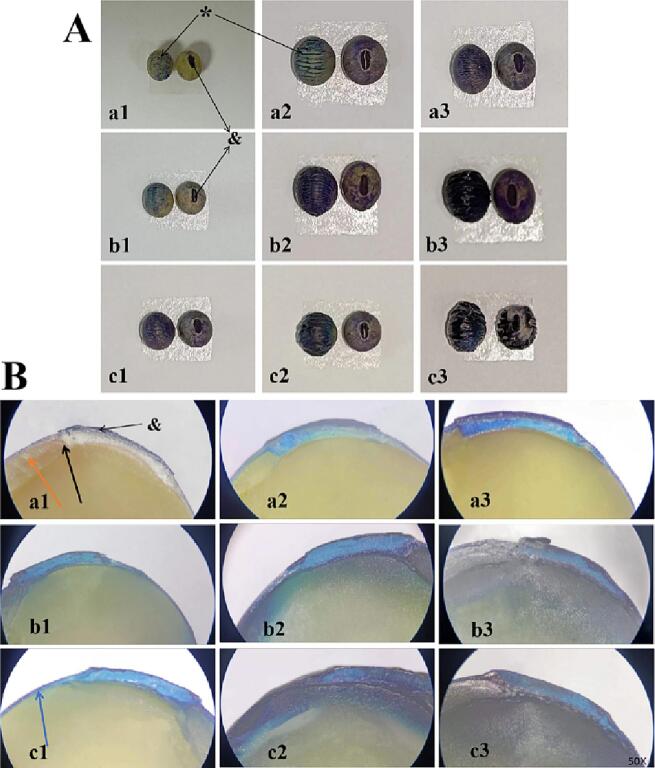
Toluidine blue stained seed exterior (A) and longitudinal seed section of under a stereomicroscope(B). Note: The letters a, b and c denote the control group, single-frequency ultrasound treatment group and double-frequency ultrasound treatment group, respectively. Numbers 1,2 and 3 represent denote toluidine blue staining times of 5 min, 20 min and 40 min respectively. *: the abaxial side, &:the hilum, black arrow: the micropyle.

Toluidine blue was applied to visualized the site and internal migration pattern of water entering the soybean. The umbilicus and the rear of the seed are the most intensely pigmented regions (Fig. 6A). The soybean has a micropyle (black arrows) adjacent to the hilum (&). According to Nakayama et al. [37], water enters the seed through the micropores (black arrows). Then it flows laterally across the embryo’s distal axis, which appears to have comparable results in the experiment (Fig. 6B, blue arrow). The action of the ultrasound seems to drive more water through the micropores. In addition, the dorsal axial side appeared significantly folded due to ultrasound, whereas it was not as pronounced in control, which may have made water enter the seeds more easily [38]. Furthermore, some research suggested that lenticular structures in soybeans are associated with seed water absorption [27], [39]. Overall, the results implied that the seeds' umbilical and dorsal axial sides might be the primary sites of water absorption. More obvious red was observed in the soybean after dual-frequency ultrasound treatment, suggesting more dyestuff enter into soybean. This means that dual-frequency ultrasound may have greater advantages for boosting water absorption in seeds, thereby accelerating subsequent sprouting.

### 3.8 Microstructure: Effect of ultrasound on the soybean seed coat

**Fig. 7:**
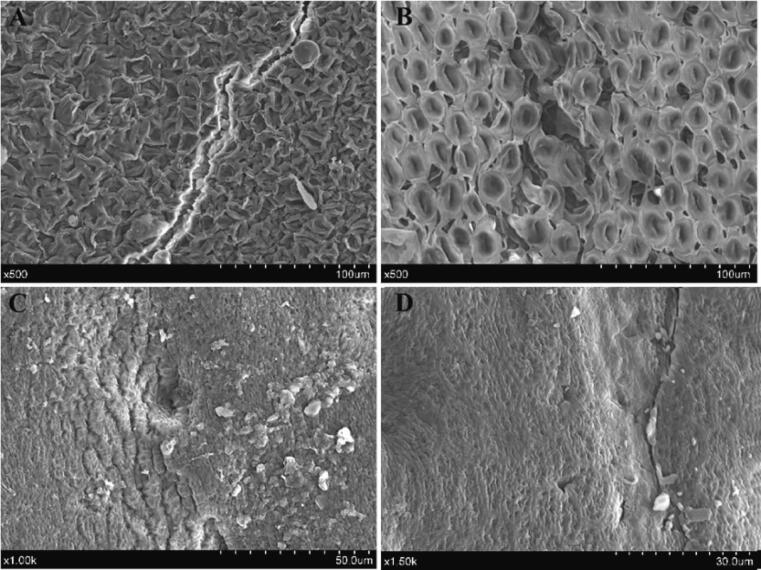
Microstructure of the soybean seed coat treated with dual-frequency ultrasound. Note: A: the surrounding surface of the hilum. B, C and D: the dorsal testa surface.

The appearance of the soybean seed coat following dual-frequency ultrasound treatment was shown in Fig. 7. Fractures were observed at the peripheral site of the hilum (A) as well as the dorsal seed coat surface (D). The ultrasonic impact was likely responsible for the appearance of holes and cell collapse (B and C) in the seed coat cells. Similar findings were observed in studies with ultrasound-treated red beans [15], where visible fissures occurred in the seed coat following ultrasound, which the cavitation and mechanical effects of ultrasound could cause.

### 3.9 Possible mechanisms

**Fig. 8:**
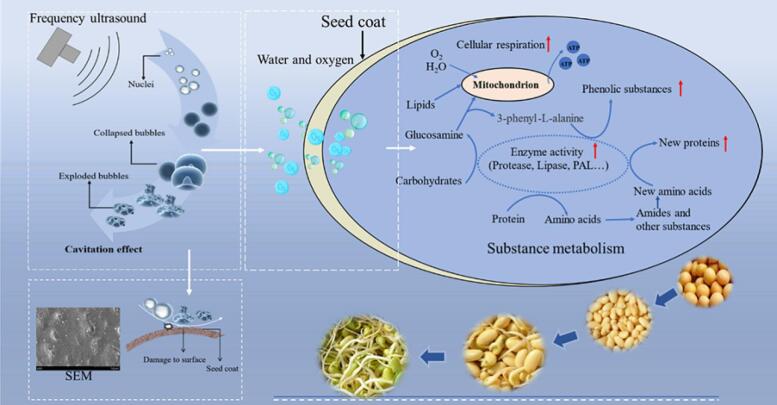
Possible mechanism diagram.

Dual-frequency ultrasound is more advantageous in accelerating seed sprouting and nutrient accumulation. The possible mechanisms (Fig. 8) were: Cavitation effect caused by ultrasonic means that, under tensile stress, bubbles in water gradually increase and collapse and burst upon the tangible interface, generating microjets and releasing energy [40]. Moreover, dual-frequency ultrasound can cause more bubble cracking resulting in more substantial energy [41]. The action of acoustic waves causes changes in the microstructure of the soybean seed coat (Fig. 6 and Fig. 7), which accelerates water absorption (Fig. 3) and enhances hydration (Fig. 4). Thus, the dormant seeds gradually restore respiration and become more vigorous (Fig. 5) after absorbing sufficient water, thereby increasing the activity of metabolism-related enzymes (Table 3 and Supplementary material). The enhanced enzymatic activity and substance metabolism within the seeds after sonication caused faster sprouting (Fig. 2), while more protein (Table 2) and phenolic substances (Fig. 3) accumulated in the later stages of sprouting.

## 4 Conclusion

In this study, dual-frequency ultrasound (20/60 kHz) treatment not only shortened sprouting timeframes, enhanced sprouting vigor, and boosted protein accumulation in soybeans during the later stages of sprouting, but also increased PAL activity, leading to higher polyphony, flavonoid accumulation, and a considerable increase in the antioxidant activity of the bean sprouts. Ultrasound treatment caused micro-cracks in the seed coat, which results in the rapid and hydrating action of water with compounds inside the seed, and the water acts as a substrate for the biochemical reactions within the seed, laying the groundwork for subsequent sprouting. This study appears to illustrate the potential of dual-frequency ultrasound in forming nutrient-rich bean sprouts. Although the promoting effect of ultrasonic mode on soybean sprouting including macronutrients accumulation, the trends of trace components like antinutritional factors and γ-aminobutyric acid still need to be further studied. Additionally, seed sprouting is a complex physiological metabolic process. Therefore, in addition to water absorption and endogenous enzymes activation, it is necessary that the underlying mechanisms should be explored from other respects like transcriptomics and proteomics in future.

## CRediT authorship contribution statement

**Jiahao Chen:** Investigation, Writing – original draft. **Feng Shao:** Methodology, Validation. **Chidimma Juliet Igbokwe:** Validation, Writing – review & editing. **Yuqing Duan:** Supervision, Project administration. **Meihong Cai:** Investigation, Validation. **Haile Ma:** Investigation, Validation. **Haihui Zhang:** Funding acquisition, Writing – review & editing.

## Declaration of Competing Interest

The authors declare that they have no known competing financial interests or personal relationships that could have appeared to influence the work reported in this paper.
