# Supplementary material for: Ultrasound treatments improve germinability of soybean seeds: The key role of working frequency
Source: Ultrason Sonochem. 2023 May 5;96:106434. doi: 10.1016/j.ultsonch.2023.106434 (PMC10197015; doi:10.1016/j.ultsonch.2023.106434)
Supplement: Supplementary Data 1 [file mmc1.docx]

Table1. Changes of activities of protease, lipase and amylase elicited with ultrasound during sprouting.

| Enzymes | Ultrasound treatment (kHz) | Sprouting time (h) | | | |
| --- | --- | --- | --- | --- | --- |
|  |  | 24 | 48 | 72 | 96 |
| Protease  (U/L) | Control | 1994.64±36.20^a^ | 2604.17±36.20^a^ | 1811.31±32.83^a^ | 1814.88±19.36^a^ |
|  | 60 | 2222.02±2.98^b^ | 2348.21±79.15^b^ | 2049.70±14.91^b^ | 1890.48±25.25^b^ |
|  | 20/60 | 2798.81±13.47^c^ | 2705.95±16.84^c^ | 2307.74±69.87^c^ | 2272.02±22.73^c^ |
| Lipase  (U/L) | Control | 2436.21±50.00^a^ | 3630.60±34.99^a^ | 2157.76±159.48^a^ | 1263.36±7.33^a^ |
|  | 60 | 2428.88±2.16^a^ | 3684.91±20.12^b^ | 2607.76±143.97^b^ | 1745.26±116.81^b^ |
|  | 20/60 | 3513.36±310.57^b^ | 4107.76±245.69^c^ | 2744.40±2.16^b^ | 2284.91±180.60c |
| Amylase  (U/L) | Control | 8554.17±529.17^a^ | 9345.83±487.50^a^ | 14454.17±179.17^a^ | 10558.33±91.67^a^ |
|  | 60 | 10258.33±508.33^b^ | 14770.83±87.50^b^ | 14233.33±366.67^a^ | 12050.00±108.33^b^ |
|  | 20/60 | 15270.83±554.17^c^ | 15456.25±4.17^b^ | 16510.42±825.00^b^ | 10454.17±45.83^a^ |

Data are presented as mean ± SD of three independent experiments. Different superscript letters within a column indicate significant differences (*p* < 0.05).
